# Supplementary material for: Circulating microRNAs as Potential Diagnostic Tools for Asthma and for Indicating Severe Asthma Risk
Source: Int J Mol Sci. 2025 Jul 11;26(14):6676. doi: 10.3390/ijms26146676 (PMC12294692; doi:10.3390/ijms26146676)
Supplement: Supplementary file 1 [file ijms-26-06676-s001.zip › ijms-3696383-supplementary changes approved.docx]

**Supplementary Table 1** Circulating miRNAs differentially expressed in the sera of asthma patients. miRNAs’ relative expressions (dCt; normalised to 6 reference miRNAs) in the sera of asthma patients (N=100) in comparison with never-asthma individuals (N=50), and mild-asthma (N=50) and severe-asthma patients (N=50) in comparison with never-asthma individuals, and the severe-asthma group in comparison with the mild-asthma cohort—severity-related miRNAs. miRNA expression was evaluated by qPCR using miRCURY LNA technology followed by GeneGlobe analysis (QIAGEN). GeneGlobe settings: fold-change threshold >1.2, Ct≤35. Initial data (fold-regulation and p-values) were obtained from GeneGlobe analysis, statistical significance (p-value*) was recalculated using the Mann–Whitney U test on individual 2⁻^ΔCt^ values to account for non-normal distribution followed by two-stage step-up method of Benjamini, Krieger, and Yekutieli false discovery rate (FDR), (Q) = 5%.

| **miRNA** | **Fold regulation** | **p-value** | **p-value*** | **FDR** |
| --- | --- | --- | --- | --- |
| **Asthma vs. Never-Asthma** |  |  |  |  |
| miR-191-5p | 1.44 | 0.000000 | 0.000001 | 0.000000 |
| miR-223-3p | 1.38 | 0.000179 | 0.000049 | 0.000292 |
| miR-197-3p | 1.33 | 0.000280 | 0.000013 | 0.000093 |
| miR-155-5p | -1.22 | 0.003573 | 0.336326 | 0.562173 |
| **Mild-Asthma vs. Never-Astnma** |  |  |  |  |
| miR-191-5p | 1.3 | 0.000032 | 0.000056 | 0.001147 |
| miR-197-3p | 1.28 | 0.007754 | 0.000710 | 0.008282 |
| **Severe-Asthma vs. Never-Asthma** |  |  |  |  |
| miR-191-5p | 1.6 | 0.000000 | 0.000000 | 0.000000 |
| miR-223-3p | 1.61 | 0.000001 | 0.000001 | 0.000007 |
| miR-197-3p | 1.37 | 0.000489 | 0.000026 | 0.000150 |
| miR-151a-3p | 1.22 | 0.024968 | 0.006008 | 0.023131 |
| miR-144-3p | -1.48 | 0.002141 | 0.006764 | 0.023437 |
| miR-125b-5p | -1.34 | 0.034275 | 0.021136 | 0.056848 |
| miR-155-5p | -1.32 | 0.006826 | 0.174644 | 0.264767 |
| miR-30a-5p | -1.28 | 0.030565 | 0.022142 | 0.056848 |
| miR-660-5p | -1.27 | 0.000300 | 0.000468 | 0.002027 |
| miR-15a-5p | -1.27 | 0.000000 | 0.000000 | 0.000000 |
| **Severe-Asthma vs. Mild-Asthma** |  |  |  |  |
| miR-223-3p | 1.37 | 0.000551 | 0.000085 | 0.005708 |
| miR-191-5p | 1.23 | 0.000027 | 0.000064 | 0.001607 |
| miR-144-3p | -1.41 | 0.011785 | 0.094333 | 0.187673 |
| miR-30a-5p | -1.4 | 0.002977 | 0.000187 | 0.002356 |
| miR-125b-5p | -1.31 | 0.031576 | 0.013337 | 0.050414 |
| miR-660-5p | -1.25 | 0.000570 | 0.001284 | 0.009707 |

**Supplementary Table 2** ROC curve analysis of asthma diagnosis and asthma severity-related miRNAs. Summary of (a) ROC analysis and (b) multiple logistic regression models. All the analyses were performed in GraphPad Prism software (version 10.0.3). The optimal cut-off values, sensitivity, and specificity were determined by calculating the Youden index. AUC—area under the ROC curve; CI—confidence interval.

(a)

| **Asthma diagnosis miRNA** | **AUC** | **Std. Error** | **95% CI** | **p-value** | **Sensitivity** | **Specificity** |
| --- | --- | --- | --- | --- | --- | --- |
| miR-223-3p | 0.7004 | 0.0460 | 0.6103–0.7905 | <0.0001 | 75% | 58% |
| miR-191-5p | 0.7988 | 0.0370 | 0.7263–0.8713 | <0.0001 | 74% | 74% |
| miR-197-3p | 0.7149 | 0.0479 | 0.6210–0.8088 | <0.0001 | 79% | 56% |
| Combined | 0.8132 | 0.03564 | 0.7433–0.8831 | <0.0001 | 76% | 72% |
| **Asthma severity miRNA** |  |  |  |  |  |  |
| miR-223-3p | 0.7238 | 0.0511 | 0.6236–0.8240 | 0.0001 | 72% | 68% |
| miR-30a-5p | 0.7132 | 0.0541 | 0.6072–0.8192 | 0.0002 | 78% | 72% |
| miR-660-5p | 0.6848 | 0.0543 | 0.5785–0.7911 | 0.0014 | 74% | 64% |
| miR-125b-5p | 0.6430 | 0.0554 | 0.5344–0.7516 | 0.0137 | 70% | 52% |
| Combined | 0.7588 | 0.04911 | 0.6625–0.8551 | <0.0001 | 78% | 64% |

(b)

| **Asthma diagnosis variable** | **Estimate** | **Std. Error** | **95% CI** | **\|Z\|** | **p-value** |
| --- | --- | --- | --- | --- | --- |
| miR-223-3p | -0.0023 | 0.0096 | -0.0213–0.0169 | 0.240 | 0.8103 |
| miR-191-5p | 1.5560 | 0.3319 | 0.9431–2.2520 | 4.690 | <0.0001 |
| miR-197-3p | 3.7000 | 2.3410 | 0.8916–8.4270 | 1.581 | 0.1140 |
| Intercept | -4.6970 | 1.0100 | -6.8400–(-2.8530) | 4.650 | <0.0001 |
| **Asthma severity variable** |  |  |  |  |  |
| miR-223-3p | 0.0154 | 0.0071 | 0.002128–0.0303 | 2.170 | 0.0300 |
| miR-30a-5p | -2.4690 | 1.7870 | -6.1800–0.9204 | 1.382 | 0.1670 |
| miR-660-5p | -4.3750 | 2.8320 | -10.1500–1.0650 | 1.538 | 0.1240 |
| miR-125b-5p | 2.2900 | 2.4530 | -2.4950–7.2840 | 0.933 | 0.3507 |
| Intercept | 0.5409 | 1.153 | -1.7100–2.8520 | 0.469 | 0.6389 |

**Supplementary Table 3** Modified asthma severity score (ASSESS). ACQ-6 score of ≥ 1.50 represents poor asthma control. Medication steps were defined by the GINA guidelines 2020. Asthma exacerbations were assessed over the preceding 12 months from WATCH study enrolment. Abbreviations: ACQ-6—asthma control questionnaire-6, FEV1—forced expiratory volume in 1 second, Salbutamol—short-acting β2-agonist (bronchodilator), ICS—inhaled corticosteroids, LTRA—leukotriene receptor antagonist, OCS—oral corticosteroids.

| **Modified Asthma Severity Score [Maximum possible score = 20]** | **Points** |
| --- | --- |
| **1) ACQ-6 Score [0**–**6 points] (30%)** | |
| <0.50 | 0 |
| 0.50–1.49 | 1 |
| 1.50–2.49 | 2 |
| 2.50–3.49 | 3 |
| 3.50–4.49 | 4 |
| 4.50–5.49 | 5 |
| 5.50–6.00 | 6 |
| **2) Current Clinic (Post-bronchodilator) Lung Function (FEV_1_ % pred.) [0**–**3 points] (15%)** | |
| ≥80.0 | 0 |
| 70.0–79.9 | 1 |
| 60.0–69.9 | 2 |
| <60.0 | 3 |
| **3) Medications [0**–**7 points] (35%)** | |
| **a) Current Medication [0**–**5 points]** | |
| No treatment | 0 |
| Step 1: Salbutamol only | 1 |
| Step 2: Low-dose ICS only or LTRA only | 2 |
| Step 3: Low-dose ICS + ≥1 controller | 3 |
| or Medium-dose ICS only |  |
| or High-dose ICS only |  |
| Step 4: Medium-dose ICS only + ≥1 controller | 4 |
| or High-dose ICS only + ≥1 controller |  |
| Step 5: High-dose ICS only + ≥2 controller | 5 |
| **b) Other Medications [0**–**2 points]** | |
| Daily/alternate day OCS or monthly corticosteroid injections | 1 |
| Current biologic | 1 |
| **4) Asthma exacerbations in past 12-months [2**–**4 points] (20%)** | |
| Prednisolone burst | 2 |
| Prednisolone burst + hospitalisation | 4 |

**Supplementary Table 4** Biological pathways affected by miRNAs differentially expressed in sera of severe-asthma patients in comparison with never-asthma group. The analysis was performed using the miRSystem tool, and pathways were chosen based on the empirical p-value cut-off of 0.05.

| Pentose and glucuronate interconversions | miR-125b-5p | miR-144-3p | miR-15a-5p |  |  |  |
| --- | --- | --- | --- | --- | --- | --- |
| Biocarta GH pathway | miR-144-3p | miR-155-5p | miR-15a-5p | miR-223-3p | miR-30a-5p |  |
| RNA polymerase I promoter opening | miR-125b-5p | miR-155-5p |  |  |  |  |
| IL4-mediated signalling events | miR-144-3p | miR-155-5p | miR-15a-5p | miR-191-5p | miR-223-3p | miR-30a-5p |
| Starch and sucrose metabolism | miR-125b-5p | miR-144-3p | miR-15a-5p |  |  |  |
| Meiotic recombination | miR-125b-5p | miR-155-5p | miR-15a-5p |  |  |  |
| Regulation of retinoblastoma protein | miR-155-5p | miR-191-5p | miR-223-3p |  |  |  |
| FOXA2 and FOXA3 transcription factor networks | miR-155-5p | miR-15a-5p | miR-191-5p |  |  |  |
| Phase II conjugation | miR-125b-5p | miR-144-3p | miR-15a-5p |  |  |  |
| Amyloids | miR-125b-5p | miR-155-5p |  |  |  |  |
| Chromosome maintenance | miR-125b-5p | miR-15a-5p | miR-30a-5p |  |  |  |
| Meiosis | miR-125b-5p | miR-155-5p | miR-15a-5p |  |  |  |
| IFN alpha beta signalling | miR-125b-5p | miR-155-5p | miR-30a-5p |  |  |  |
| Antigen processing ubiquitination proteasome degradation | miR-125b-5p | miR-155-5p | miR-15a-5p | miR-30a-5p |  |  |
| MHC I-mediated antigen processing presentation | miR-125b-5p | miR-155-5p | miR-15a-5p | miR-30a-5p |  |  |
| Cytokine signalling in immune system | miR-125b-5p | miR-144-3p | miR-155-5p | miR-15a-5p | miR-223-3p | miR-30a-5p |
