# Supplementary material for: Circulating microRNAs as Potential Diagnostic Tools for Asthma and for Indicating Severe Asthma Risk
Source: Int J Mol Sci. 2025 Jul 11;26(14):6676. doi: 10.3390/ijms26146676 (PMC12294692; doi:10.3390/ijms26146676)

**Supplementary Figure 1** Quartile Stratification of m-ASSESS among Severe Asthma Patients

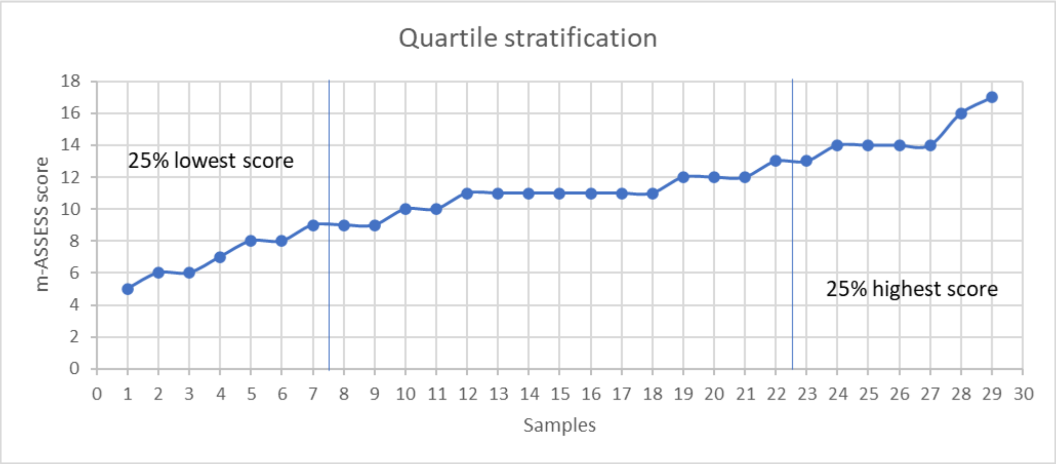

**Supplementary Figure 2** Pilot study outcome showing miRNAs differentially expressed in the sera of severe-asthma (SA) patients in comparison with mild-asthma (MA) and never-asthma (NA) individuals [M.A. Kyyaly et al. Circulating miRNAs—A potential tool to identify severe asthma risk? *Clin Transl Allergy*. 2021;e12040].

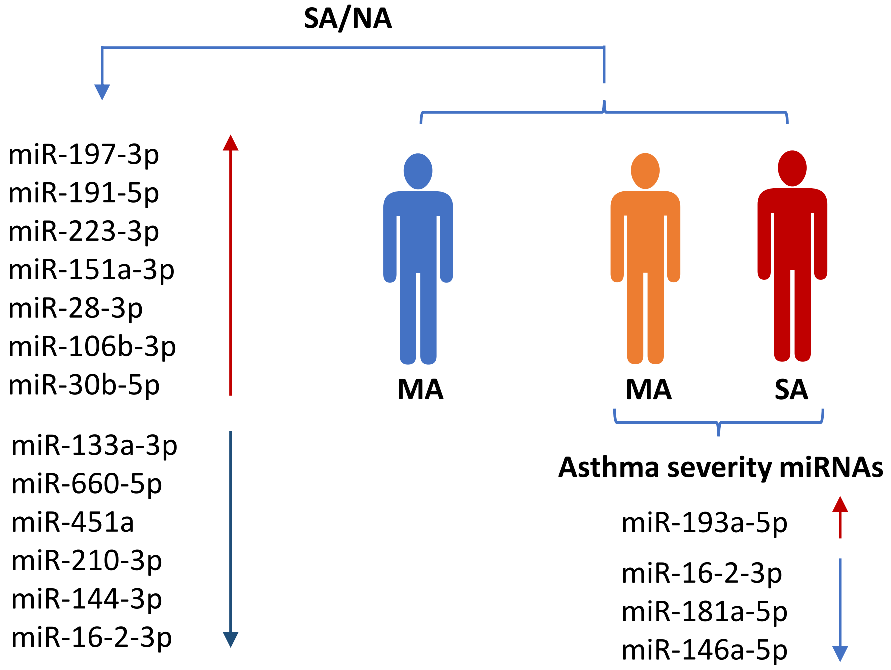

Supplement: Supplementary file 1 [file ijms-26-06676-s001.zip › Figures_SuppInfo REVISED V2.pdf]
